# Complex assembly, crystallization and preliminary X-ray crystallographic analysis of the human Rod–Zwilch–ZW10 (RZZ) complex

**DOI:** 10.1107/S2053230X15004343

**Published:** 2015-03-20

**Authors:** Anika Altenfeld, Sabine Wohlgemuth, Annemarie Wehenkel, Ingrid R. Vetter, Andrea Musacchio

**Affiliations:** a Department of Mechanistic Cell Biology, Max Planck Institute of Molecular Physiology, Otto Hahn Strasse 11, 44227 Dortmund, Germany; b Department ‘Genotoxic Stress and Cancer’, Institut Curie, CNRS UMR 3348/INSERM U1005, Bâtiment 110, Centre Universitaire, 91405 Orsay CEDEX, France; c Center for Medical Biotechnology, University of Duisburg-Essen, Universitätstrasse 1, 45141 Essen, Germany

**Keywords:** spindle-assembly checkpoint, cell division, mitosis, RZZ complex, Rod, Zwilch, ZW10, kinetochore, Ndc80, Mis12, Knl1, KMN network

## Abstract

The spindle-assembly checkpoint (SAC) monitors kinetochore–microtubule attachment during mitosis. In metazoans, the three-subunit Rod–Zwilch–ZW10 (RZZ) complex is a crucial SAC component that interacts with additional SAC-activating and SAC-silencing components, including the Mad1–Mad2 complex and cytoplasmic dynein. The RZZ complex contains two copies of each subunit and has a predicted molecular mass of ∼800 kDa. Given the low abundance of the RZZ complex in natural sources, its recombinant reconstitution was attempted by co-expression of its subunits in insect cells. The RZZ complex was purified to homogeneity and subjected to systematic crystallization attempts. Initial crystals containing the entire RZZ complex were obtained using the sitting-drop method and were subjected to optimization to improve the diffraction resolution limit. The crystals belonged to space group *P*3_1_ (No. 144) or *P*3_2_ (No. 145), with unit-cell parameters *a* = *b* = 215.45, *c* = 458.7 Å, α = β = 90.0, γ = 120.0°.

## Abstract 2

The 800 kDa complex of the human Rod, Zwilch and ZW10 proteins (the RZZ complex) was reconstituted in insect cells, purified, crystallized and subjected to preliminary X-ray diffraction analysis.

## 1. Introduction   

The goal of cell division (mitosis) is to create progeny with identical copies of the genome. Chromosomes interact with the mitotic spindle *via* kinetochores, assemblies of ∼30 core subunits arranged around a unique chromosomal locus named the centromere (Santaguida & Musacchio, 2009 ▶). During mitosis, kinetochores recruit and control the activity of the spindle-assembly checkpoint (SAC), a surveillance mechanism that monitors chromosome attachment to prevent exit from mitosis until all chromosomes are properly oriented on the mitotic spindle (Lara-Gonzalez *et al.*, 2012 ▶).

The three-subunit Rod–Zwilch–ZW10 (RZZ) complex is crucial for SAC function in metazoans (Karess, 2005 ▶; Scaërou *et al.*, 2001 ▶; Williams *et al.*, 2003 ▶; Chan *et al.*, 2000 ▶; Basto *et al.*, 2004 ▶). After becoming recruited to unattached kinetochores in late prophase/early pro-metaphase, the RZZ complex contributes to recruiting the Mad1–Mad2 complex, a crucial SAC component (Buffin *et al.*, 2005 ▶; Kops *et al.*, 2005 ▶). The RZZ complex also recruits the minus end-directed microtubule motor dynein–dynactin to kinetochores, promoting SAC silencing upon microtubule attachment (Basto *et al.*, 2004 ▶; Starr *et al.*, 1998 ▶; Howell *et al.*, 2001 ▶; Wojcik *et al.*, 2001 ▶).

In humans, Rod (2209 residues), ZW10 (672 residues) and Zwilch (591 residues), the three subunits of the RZZ complex, have approximate predicted molecular masses of 250, 89 and 67 kDa, respectively. Ultracentrifugation and size-exclusion chromatography (SEC) experiments suggested that the RZZ complex might have an overall molecular mass of ∼800 kDa, compatible with a 2:2:2 stoichiometry of the three constitutive subunits (Çivril *et al.*, 2010 ▶; Williams *et al.*, 2003 ▶).

We have previously reported the crystal structure of human Zwilch (PDB entry 3if8) and demonstrated that it represents a new fold (Çivril *et al.*, 2010 ▶). We also demonstrated that Zwilch interacts directly with a 350-residue segment in the N-terminal region of Rod predicted to fold as a β-propeller (Çivril *et al.*, 2010 ▶). Despite these advances, the overall structural organization of the RZZ complex, and how it translates into its function, remains unknown. To make new inroads, we biochemically reconstituted the human RZZ complex by co-expression of its subunits in insect cells. After purification by affinity and size-exclusion chromatography (SEC), the RZZ complex appeared to be homogenous and its subunits were represented stoichiometrically. We report the successful crystallization of the RZZ complex and describe the steps required for improvement of the crystal quality.

## 2. Materials and methods   

### 2.1. RZZ complex production   

The DNA sequences of human Zwilch and Rod were subcloned into a pACEbac1 or pFL expression vector (ATG Biosynthetics, Merzhausen, Germany). Expression of Zwilch with two His residues at its N-terminus considerably enhanced its expression levels. Rod was expressed with an N-terminal hexahistidine tag with a linker and a cleavage site for TEV protease, leading to 18 additional residues between the hexahistidine tag and the Rod sequence (shown as underlined characters in Supplementary Table S1). For the pACEbac1 and pFL constructs, bacmid recombination and virus production were carried out as described by Bieniossek *et al.* (2008 ▶). To express the entire RZZ complex, 500 ml of TnaO38 cells (Hashimoto *et al.*, 2010 ▶) at a cell density of 10^6^ cells ml^−1^ were co-infected with the pACEbac1_ZW10, pACEbac1_His_2__Zwilch and pFL_His_6__Rod viruses. Cells were harvested by centrifugation for 20 min at 500*g*. The pellet was resuspended in 100 ml lysis buffer [50 m*M* HEPES pH 8.5, 200 m*M* NaCl, 5% glycerol, 5 m*M* β-mercaptoethanol, 1 m*M* phenylmethylsulfonyl fluoride (PMSF)], disrupted by sonication and cleared by centrifugation at 100 000*g* for 45 min. The cleared lysate was loaded onto a 5 ml Ni–NTA column (GE Healthcare) equilibrated with loading buffer [50 m*M* HEPES pH 8.5, 200 m*M* NaCl, 5% glycerol, 5 m*M* β-mercaptoethanol, 20 m*M* imidazole] at a flow rate of 1.5 ml min^−1^ using a peristaltic pump. For optimal yields, the flowthrough was loaded a second time. The column was washed with 500 ml loading buffer. Elution was either performed with imidazole or by PreScission cleavage on the column. The protein was further purified by size-exclusion chromatography using a Superose 6 10/300 column equilibrated with 25 m*M* HEPES pH 8.5, 250 m*M* NaCl, 4 m*M* TCEP. Fractions containing the RZZ complex were pooled and concentrated to 5–10 mg ml^−1^ using an Amicon Ultra MWCO 10 000 concentrator, flash-cooled in aliquots of 20 µl volume and stored at −80°C. Macromolecule-production information is summarized in Table 1 ▶.

### 2.2. Crystallization   

Initial crystals were obtained using condition No. 69 (1 *M* ammonium sulfate, 0.1 *M* MES pH 6.5) of The ProComplex Suite (Qiagen, Hilden, Germany) by the sitting-drop vapour-diffusion method as described in Table 2 ▶. Table 2 ▶ describes initial optimization attempts, including interventions aiming to reduce the potential biochemical heterogeneity of the sample. A first attempt was the removal of the hexahistidine tag from Rod using PreScission protease during elution from an Ni–NTA column. In addition, evidence from mass-spectrometric analyses that the RZZ complex is phosphorylated during expression in insect cells prompted us to attempt dephos­phorylation using λ-phosphatase (data not shown). As post-crystallization treatments, we tried to anneal the crystals by briefly interrupting the liquid-nitrogen stream that usually keeps the crystals at 100 K. Data-collection experiments at room temperature were also performed. As an additional approach, dehydration of the crystals was attempted by either serial addition of glycerol to the reservoir solution or by increasing the precipitant concentration in 2–5% steps every 2 d.

### 2.3. Data collection and processing   

In order to choose a suitable cryoprotectant, crystals were harvested in the reservoir buffer and soaked directly or serially (in 2–5% steps) in reservoir buffer supplemented with 5–20% ethylene glycol, glycerol or PEG 400, flash-cooled in liquid nitrogen and tested in a cryo-beam for ice rings. Diffraction data were collected at 100 K on beamline X10SA at the Swiss Light Source (SLS), Villigen, Switzerland using a PILATUS 6M detector at a wavelength of 0.9789 Å. Data were processed and scaled using *XDS* and *XSCALE* (Kabsch, 2010 ▶).

## 3. Results and discussion   

**Figure 1:**
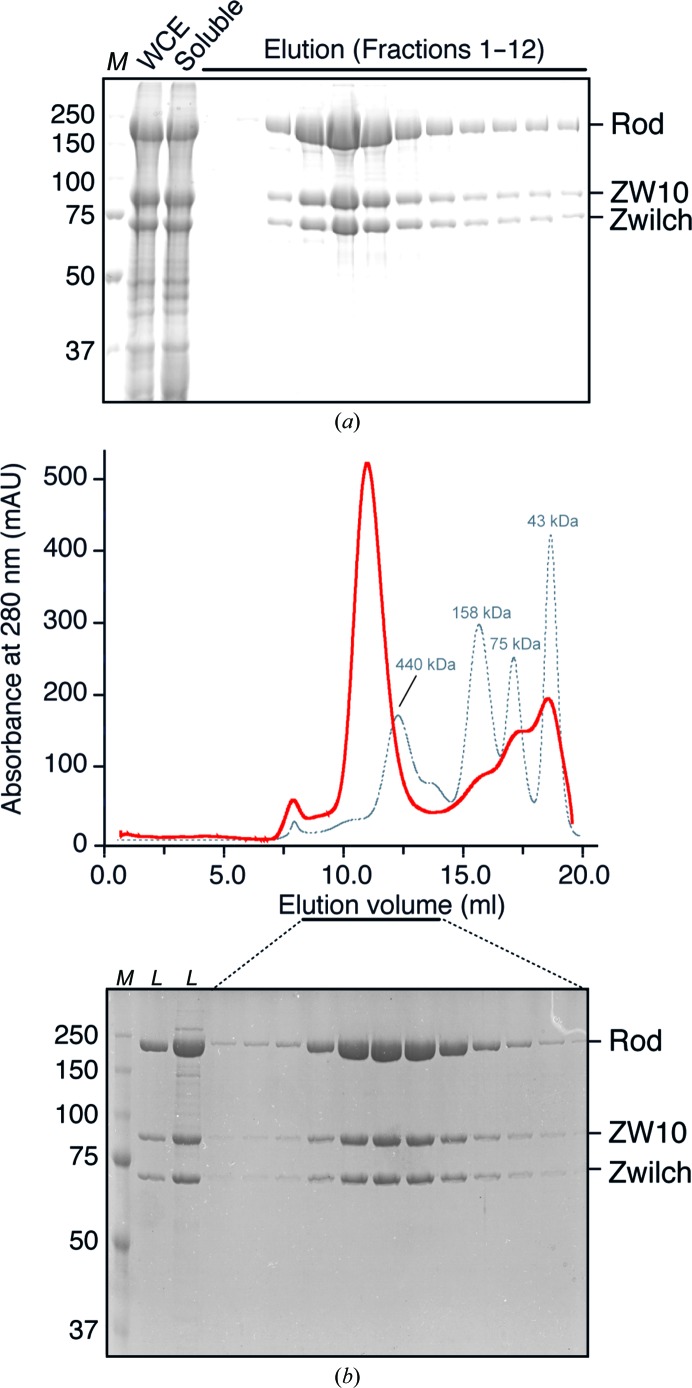
Documentation of the purification procedure. (*a*) SDS–PAGE separations of samples representing whole cell extracts (WCE), the soluble fraction after lysate clearance by ultracentrifugation and fractions of elution from immobilized metal-chelating chromatography. Lane *M* contains molecular-weight marker (labelled in kDa). (*b*) After pooling and concentration, the sample was further separated by size-exclusion chromatography on a Superose 6 10/300 column. The elution profiles of globular markers of the indicated molecular mass are shown. The elution profile and corresponding protein content are shown. The lanes marked *L* contain two concentrations of sample loaded onto the column. Lane *M* contains molecular-weight marker (labelled in kDa).

**Figure 2:**
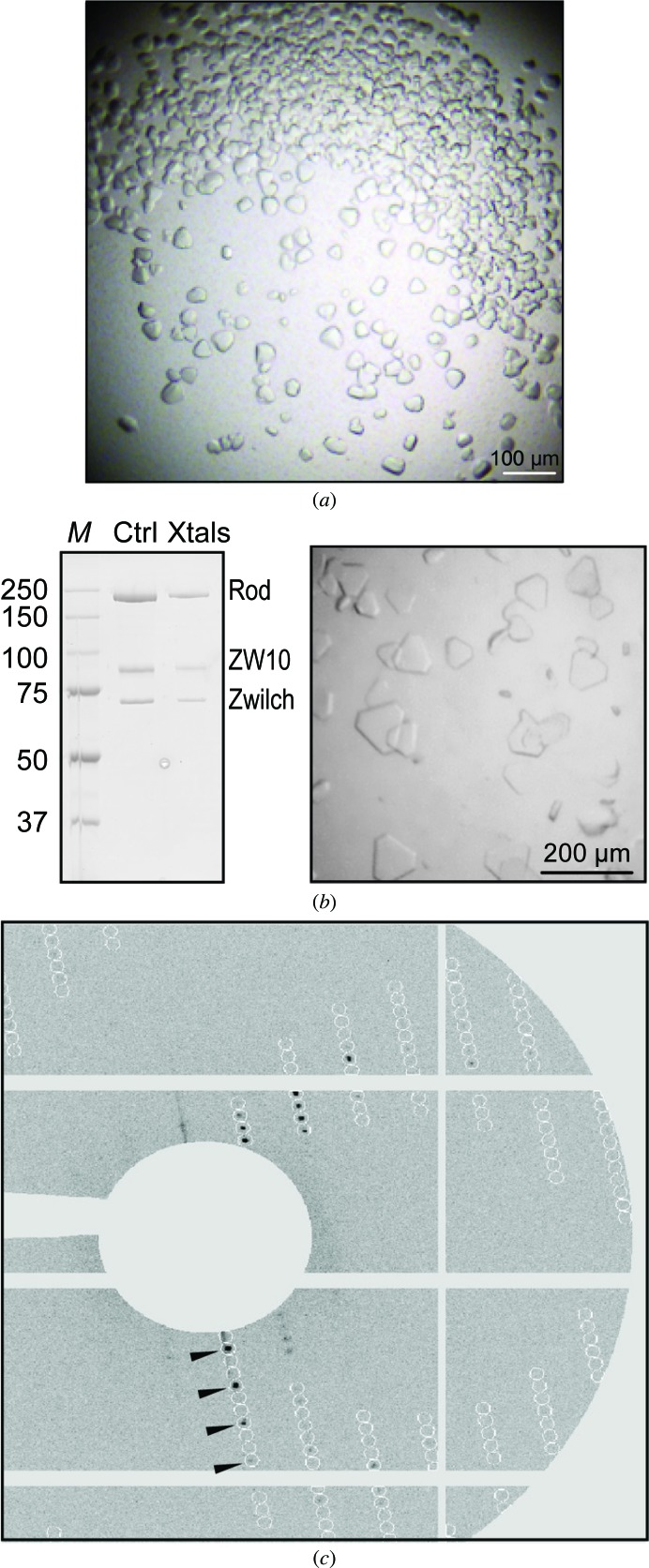
Crystallization of the RZZ complex and diffraction image. (*a*) Initial crystals of the RZZ complex. (*b*) Dissolved crystals (Xtals) contain all three subunits in apparently unaltered form with respect to the original sample (Ctrl). (*c*) Crystals after optimization. (*d*) Diffraction pattern showing diffraction peaks (indicated by black arrowheads) alternating with two systematic absences along the *c* axis.

**Figure 3:**
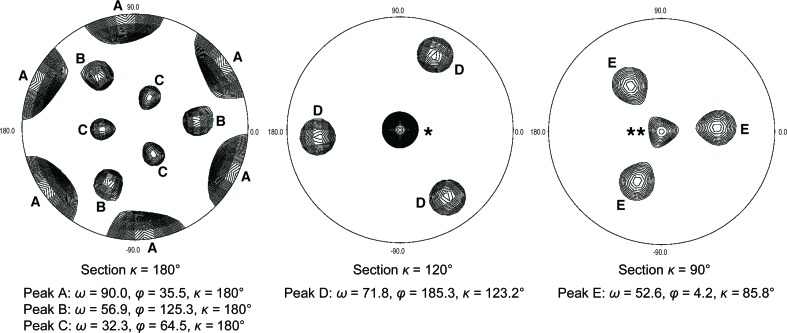
The self-rotation function is shown for the indicated sections. Polar angles of the main noncrystallographic symmetry axes are reported. The twofold axes in the κ = 180° section may be generated by internal twofold symmetry of the RZZ complex (which is likely to form a 2:2:2 hexamer) as well as the likely presence of two RZZ complexes in the asymmetric unit of the crystal. The single asterisk in the central panel marks the crystallographic threefold axis. The double asterisk in the right panel marks the ‘bleed-through’ of the threefold axis in the 90° section. Peaks B and D and peaks C and E are symmetry-related. The relative heights of the peaks (origin peak = 100) were as follows: peak A, 98.8; peak B, 56.4; peak C, 50.2; ‘bleed-through’ peak of the crystallographic threefold axis, 49.

The recombinant full-length RZZ complex was purified in a two-step approach using a nickel resin affinity column followed by size-exclusion chromatography (Figs. 1 ▶ *a* and 1 ▶ *b*). After the second step of the purification procedure the RZZ complex was >98% pure based on SDS–PAGE analysis (Fig. 1 ▶ *b*). The initial crystallization screening produced crystals in a single condition (1 *M* ammonium sulfate, 0.1 *M* MES pH 6.5). The strategy followed for the optimization of these initial crystals is described in Table 2 ▶. Crystals (Fig. 2 ▶ *a*) grown against a reservoir buffer consisting of 380 m*M* ammonium sulfate, 0.1 *M* MES pH 6.3 that resulted in the data set described in this article were washed extensively and analysed by SDS–PAGE analysis. This revealed three bands corresponding to the three individual proteins that comprise the pure RZZ complex devoid of apparent signs of degradation (Fig. 2 ▶ *b*). Crystals were soaked in cyroprotecting solution consisting of 0.5 *M* ammonium sulfate, 20% ethylene glycol and a data set was collected at the SLS synchrotron, Villigen, Switzerland (Table 3 ▶). The crystals showed a clean diffraction pattern to 18 Å resolution with additional reflections extending to a 14 Å resolution limit. Processing of the diffraction data revealed symmetry and systematic absences typical of the trigonal space groups *P*3_1_ or *P*3_2_ (Fig. 2 ▶ *d*). Judging from the unit-cell dimensions, two RZZ complexes, each consisting of two heterotrimers, are likely to fit into the asymmetric unit, with a Matthews parameter of 3.8 Å^3^ Da^−1^ corresponding to a solvent content of 68% (Alternatively, with three heterotrimers in the asymmetric unit, the Matthews parameter would be expected to be 2.56 Å^3^ Da^−1^ and the solvent content 52%.) The largest peaks in the self-rotation function (Fig. 3 ▶) indicate the presence of noncrystallographic twofold axes orthogonal to the crystallo­graphic threefold that are likely to relate the two RZZ complexes in the asymmetric unit. Two additional twofold symmetry axes might correspond to internal symmetry elements of one RZZ complex, perhaps reflecting the 2:2:2 stoichiometry.

We report for the first time the recombinant reconstitution of the RZZ complex and demonstrate that it can be obtained in high yield and in a grade suitable for crystallization. Given the size and molecular complexity of the RZZ complex, this is an exciting achievement. It paves the way for biochemical and structural characterization of the complex. Our future efforts will be directed towards the optimization of crystal growth and post-crystallization processing to improve the diffraction limit of our crystals.

## Supplementary Material

Supplementary Table S1.. DOI: 10.1107/S2053230X15004343/pg5052sup1.pdf

## Figures and Tables

**Table 1:** Composition of the human RZZ complex and expression strategy Restriction sites in the forward and reverse primers are underlined.

	Rod	ZW10	Zwilch
Source organism	*Homo sapiens*	*Homo sapiens*	*Homo sapiens*
DNA source	cDNA	cDNA	cDNA
Forward primer	GCGCGGATCCATGTGGAACGATATTGAACTGC	CGCGGATCCATGGCCTCGTTCGTGACAGAAG	CGCGGATCCATGCATCACATGTGGGAGCGGCTGAACTGC
Reverse primer	GCGCGTCGACTTACGATAATCCACTAAGAAACATCTTCAG	CGCGTCGACTTATCATTTAATTTTAGCAAGGGCAGCTGC	ACGCGTCGACTCATTACTTGAAATGCACCTGGCTGC
Cloning vector	pFL	pACEbac1	pACEbac1
Expression vector	pFL_His_6__Rod	pACEbac1_ZW10	pACEbac1_His_2__Zwilch
Expression host	Tnao38 (cabbage looper)	Tnao38 (cabbage looper)	Tnao38 (cabbage looper)
Complete amino-acid sequence of the construct produced	See Supplementary Table S1	See Supplementary Table S1	See Supplementary Table S1

**Table 2:** Crystallization conditions and initial optimization

Method	Initial screening	Optimization screening	Additive screening	Seeding	*In situ* proteolysis
Plate type	96-well	24-well	96-well	24-well	96-well, 24-well
Temperature (K)	277.15, 293.15	277.15, 285.15, 293.15	293.15	293.15	285.15, 293.15
Protein concentration (mgml^1^)	510	510	510	510	510
Buffer composition of protein solution	25m*M* HEPES pH 8.5, 250m*M* NaCl, 2m*M* TCEP	Unchanged	Unchanged	Unchanged	Unchanged
Composition of reservoir solution/screening kit	The JSCG Core IIV, ProComplex, Anions, Cations, Cryo, PEGs, PEGs II and AmSO_4_ Suites (Qiagen)	Buffer, pH, salt and glycerol were varied systematically around condition 69 of the The ProComplex Suite as well as other conditions identified by initial screening of RZZ complex devoid of His_6_ tag and/or dephosphorylated	Additive Screen (Hampton Research) was applied to various conditions obtained after initial optimization	In drops with various conditions obtained after initial optimization but with precipitant concentrations reduced to 75% of the original condition. Seed-stock preparation: crystals from a similar condition were typically mixed with 100200l reservoir solution and smashed by adding a glass bead and vortexing followed by serial streaking with a cat whisker	Based on various conditions obtained after initial optimization, performed as in Dong *et al.* (2007 ▶). Trypsin and elastase dilutions from 1:100 to 1:20000
Volume and ratio of drop	300nl, 1:1, 1:2, 2:1	18l, 1:1, 1:2, 2:1	300nl, 1:1, 1:2, 2:1	12l, 1:1, 1:2, 2:1	300nl, 1:1, 1:2, 2:1 for 96-well; 1l, 1:1, 1:2, 2:1 for 24-well
Volume of reservoir (l)	70	500	70	500	70 for 96-well, 500 for 24-well

**Table 3:** Data collection and processing Values in parentheses are for the outer shell.

Diffraction source	X10SA, SLS
Wavelength ()	0.9789
Temperature (K)	100
Detector	Pilatus 6M
Crystal-to-detector distance (mm)	1080
Rotation range per image ()	0.25
Total rotation range ()	180
Exposure time per image (s)	0.25
Space group	*P*3_1_ [No. 144] or *P*3_2_ [No. 145]
Unit-cell parameters (, )	*a* = *b* = 215.4, *c* = 458.7, = = 90, = 120
Mosaicity ()	0.213
Resolution range ()	6018
Total No. of reflections	10804
No. of unique reflections	2073
Completeness (%)	94.8 (100)
Multiplicity	5.21 (5.47)
*I*/(*I*)	8.8 (2.29)
*R*_meas_ (%)	14.5 (96.3)
CC_1/2_	99.5 (73.4)
