# Supplementary material for: Complex assembly, crystallization and preliminary X-ray crystallographic analysis of the human Rod–Zwilch–ZW10 (RZZ) complex
Source: Acta Crystallogr F Struct Biol Commun. 2015 Mar 20;71(Pt 4):438–42. doi: 10.1107/S2053230X15004343 (PMC4388180; doi:10.1107/S2053230X15004343)
Supplement: Supplementary file 1 [file f-71-00438-sup1.pdf]

STRUCTURAL BIOLOGY COMMUNICATIONS

Volume 71 (2015)

Supporting information for article:

Complex assembly, crystallization and preliminary X-ray crystallographic analysis of the human Rod–Zwilch–ZW10 (RZZ) complex

Anika Altenfeld, Sabine Wohlgemuth, Annemarie Wehenkel, Ingrid R. Vetter and Andrea Musacchio

Table S1

Complete amino acid sequence of constructs produced. In Rod, the sequence underlined separates the His_6 tag from the natural N-terminus of the protein

Rod	ZW10	Zwilch
MSYYHHHHHHDYDIPTTENLYFQGAMG<br>SMWNDIELLTNDDTGSGYLSVGSRKEH<br>GTALYQVDLLVKISSEKASLNPKIQAC<br>SLSDGFIIVADQSVILLDSICRSLQLH<br>LVFDTEVDVVGLCQEGKFLLVGERSGN<br>LHLIHVTSKQTLLTNAFVQKANDENRR<br>TYQNLVIEKDGSNEGTYYMLLLTYSGF<br>FCITNLQLLKIQQAIENVDFSTAKKLQ<br>GQIKSSFISTENYHTLGCLSLVAGDLA<br>SEVPVIIGGTGNCAFSKWEPDSSKKGM<br>TVKNLIDAEIIKGAKKFQLIDNLLFVL<br>DTDNVLSLWDIYTLTPVWNWPSLHVEE<br>FLLTTEADSPSSVTWQGITNLKLIALT<br>ASANKKMKNLMVYSLPTMEILYSLEVS<br>SVSSLVQTGISTDTIYLLEGVCKNDPK<br>LSEDSVSVLVLRCLTEALPENRLSRLL<br>HKHRFAEAESFAIQFGLDVELVYKVKS<br>NHILEKLALSSVDASEQTEWQQLVDDA<br>KENLHKIQDDEFVVNYCLKAQWITYET<br>TQEMLNYAKTRLLKKEDKTALIYSDGL<br>KEVLRAHAKLTTFYGAFGPEKFSGSSW<br>IEFLNNEDDLKDIFLQLKEGNLVCAQY<br>LWLRHRANFESRFDVKMLESLLNSMSA<br>SVSLQKLCPWFKNDVIPFVRRTVPEGQ<br>IILAKWLEQAARNLELTDKANWPENGL<br>QLAEIFFTAEKTDELGLASSWHWISLK<br>DYQNTEEVCQLRTLVNNLRELITLHRK<br>YNCKLALSDFEKENTTTIVFRMFDKVL<br>APELIPSILEKFIRVYMREHDLQEEEL<br>LLLYIEDLLNRCSSKSTSLFETAWEAK<br>AMAVIACLSDTDLIFDAVLKIMYAAVV<br>PWSAAVEQLVKQHLEMDHPKVKLLQES<br>YKLMEMKKLLRGYGIREVNLLNKEIMR<br>VVRYILKQDVPSSLEDALKVAQAFMLS<br>DDEIYSLRIIDLIDREQGEDCLLLLKS<br>LPPAEAEKTAERVIIWARLALQEEPDH<br>SKEGKAWRMSVAKTSVDILKILCDIQK<br>DNLQKKDECEEMLKLFKEVASLQENFE<br>VFLSFEDYSNSSLVADLREQHIKAHEV<br>AQAKHKPGSTPEPIAAEVRSPSMESKL<br>HRQALALQMSKQELEAELTLRALKDGN<br>IKTALKKCSDLFKYHCNADTGKLLFLT<br>CQKLCQMLADNVPVTVPVGLNLPSMIH<br>DLASQAATICSPDFLLDALELCKHTLM<br>AVELSRQCQMDDCGILMKASFGTHKDP<br>YEEWSYSDFFSEDGIVLESQMVLPVIY<br>ELISSLVPLAESKRYPLESTSLPYCSL<br>NEGDGLVLPVINSISALLQNLQESSQW<br>ELALRFVVGSFGTCLQHSVSNFMNATL<br>SEKLFGETTLVKSRHVVMELKEKAVIF<br>IRENATTLLHKVFNCRLVDLDLALGYC<br>TLLPQKDVFENLWKLIDKAWQNYDKIL<br>AISLVGSELASLYQEIEMGLKFRELST<br>DAQWGIRLGKLGISFQPVFRQHFLTKK<br>DLIKALVENIDMDTSLILEYCSTFQLD<br>CDAVLQLFIETLLHNTNAGQGQGDASM<br>DSAKRRHPKLLAKALEMVPLLTSTKDL	MASFVTEVLAHSGRLEKEDLGTRISRL<br>TRRVEEIKGEVCNMISKKYSEFLPSMQ<br>SAQGLITQVDKSEDIDLLKSRIESEVR<br>RDLHVSTGEFTDLKQQLERDSVVLSLL<br>KQLQEFSTAIEEYNCALTEKKYVTGAQ<br>RLEEAQKCLKLLKSRKCFDLKILKSLS<br>MELTIQKQNILYHLGEEWQKLIVWKFP<br>PSKDTSSLESYLQTELHLYTEQSHKEE<br>KTPMPPISSVLLAFSVLGELHSKLKSF<br>GQMLLKYILRPLASCPSLHAVIESQPN<br>IVIIRFESIMTNLEYPSPSEVFTKIRL<br>VLEVLQKQLLDLPLDTDLENEKTSTVP<br>LAEMLGDMIWEDLSECLIKNCLVYSIP<br>TNSSKLQQYEEIIQSTEEFENALKEMR<br>FLKGDTTDLLKYARNINSHFANKKCQD<br>VIVAARNLMTSEIHNTVKIIPDSKINV<br>PELPTPDEDNKLEVQKVSNTQYHEVMN<br>LEPENTLDQHSFSLPTCRISESVKKLM<br>ELAYQTLLEATTSSDQCAVQLFYSVRN<br>IFHLFHDVVPTYHKENLQKLPQLAAIH<br>HNNCMYIAHHLLTLGHQFRLRLAPILC<br>DGTATFVDLVPGFRRLGTECFLAQMRA<br>QKGELLERLSSARNFSNMDDEENYSAA<br>SKAVRQVLHQLKRLGIVWQDVLPVNIY<br>CKAMGTLLNTAISEVIGKITALEDIST<br>EDGDRLYSLCKTVMDEGPQVFAPLSEE<br>SKNKKYQEEVPVYVPKWMPFKELMMML<br>QASLQEIGDRWADGKGPLAAAFSSSEV<br>KALIRALFQNTERRAAALAKIK	MHHMWERLNCAAEDFYSRLLQKFNEEK<br>KGIRKDPFLYEADVQVQLISKGQPNPL<br>KNILNENDIVFIVEKVPLEKEETSHIE<br>ELQSEETAISDFSTGENVGPLALPVGK<br>ARQLIGLYTMAHNPNMTHLKINLPVTA<br>LPPLWVRCDSSDPEGTCWLGAELITTN<br>NSITGIVLYVVSCKADKNYSVNLENLK<br>NLKKRHHLSTVSKGFAQYELFKSSALD<br>DTITASQTAIALDISWSPVDEILQIPP<br>LSSTATLNIKVESGEPRPLNHLYRELK<br>FLLVLADGLRTGVTEWLEPLEAKSAVE<br>LVQEFLNDLNKLDGFGDSTKKDTEVET<br>LKHDTAAVDRSVKRLFKVRSDLDFAEQ<br>LWCKMSSSVISYQDLVKCFTLIIQSLQ<br>RGDIQPWLHSGSNSLLSKLIHQSYHGT<br>MDTVSLSGTIPVQMLLEIGLDKLKKDY<br>ISFFIGQELASLNHLEYFIAPSVDIQE<br>QVYRVQKLHILEILVSCMPFIKSQHEL<br>LFSLTQICIKYYKQNPLDEQHIFQLPV<br>RPTAVKNLYQSEKPQKWRVEIYSGQKK<br>IKTVWQLSDSSPIDHLNFHKPDFSELT<br>LNGSLEERIFFTNMVTCSQVHFK

VISLSGILHKLDPYDYEMIEVVLKVIE<br>RADEKITNININQALSILKHLKSYRRI<br>SPPVDLEYQYMLEHVITLPSAAQTRLP<br>FHLIFFGTAQNFWKILSTELSEESFPT<br>LLLISKLMKFSLDTLYVSTAKHVFEKK<br>LKPKLLKLTQAKSSTLINKEITKITQT<br>IESCLLSIVNPEWAVAIAISLAQDIPE<br>GSFKISALKFCLYLAERWLQNIPSQDE<br>KREKAEALLKKLHIQYRRSGTEAVLIA<br>HKLNTEEYLRVIGKPAHLIVSLYEHPS<br>INQRIQNSSGTDYPDIHAAAKEIAEVN<br>EINLEKVWDMLLEKWLCPSTKPGEKPS<br>ELFELQEDEALRRVQYLLLSRPIDYSS<br>RMLFVFATSTTTTLGMHQLTFAHRTRA<br>LQCLFYLADKETIESLFKKPIEEVKSY<br>LRCITFLASFETLNIPITYELFCSSPK<br>EGMIKGLWKNHSHESMAVRLVTELCLE<br>YKIYDLQLWNGLLQKLLGFNMIPYLRK<br>VLKAISSIHSLWQVPYFSKAWQRVIQI<br>PLLSASCPLSPDQLSDCSESLIAVLEC<br>PVSGDLDLIGVARQYIQLELPAFALAC<br>LMLMPHSEKRHQQIKNFLGSCDPQVIL<br>KQLEEHMNTGQLAGFSHQIRSLILNNI<br>INKKEFGILAKTKYFQMLKMHAMNTNN<br>ITELVNYLANDLSLDEASVLITEYSKH<br>CGKPVPPDTAPCEILKMFLSGLS		
